# Differences of medically unexplained symptoms among patients of different ages and sexes in the psychological clinic of a general hospital and the influencing factors of MUS: A cross-sectional study

**DOI:** 10.3389/fpsyt.2022.930212

**Published:** 2022-08-04

**Authors:** Jie Zhang, Yu Pan, Jiangyue Hong, Hong Guo, Mengyu Wang, Xiaolei Liu, Yanbin Dong, Dejun Wang, Lu Liu, Shuping Tan, Ronghuan Jiang

**Affiliations:** ^1^Beijing HuiLongGuan Hospital, Peking University HuiLongGuan Clinical Medical School, Beijing, China; ^2^Department of Psychological Medicine, The First Medical Center, Chinese PLA (People's Liberation Army) General Hospital, Beijing, China

**Keywords:** depression, medically unexplained symptoms, stress, psychological, resilience, social support

## Abstract

**Objective:**

To analyse differences in sex, age, depression, insomnia, psychological stress, resilience, and perceived social support among patients with medically unexplained symptoms (MUS) in a psychological clinic of a general hospital, and to explore the influencing factors of MUS.

**Methods:**

This is a cross-sectional study. Seven hundred forty-six first-time patients were assessed with the integrated psychosomatic comprehensive evaluation system (IPS) to evaluate their MUS, depression, insomnia, psychological stress, resilience, and perceived social support. The psychological characteristics were compared with regard to sex and age group (<25 years, low age group; 26–44 years, middle age group; >45 years, high age group). The relationships between age and MUS were explored, and how psychological stress affects MUS was analyzed using the mediator effect model.

**Results:**

Different age groups had significant differences in sex, MUS, depression, psychological stress, resilience, and perceived social support. In further pairwise comparison, no significant difference existed in depression, psychological stress, resilience and perceived social support in the middle and low age groups, depression and psychological stress were higher than those in the high age group, resilience and perceived social support were lower than those of the high age group. MUS were higher in the middle age group than in the low age group. No significant difference existed between the two groups and the high age group. Age, severity of MUS, and perceived social support were significantly different between the sexes. Differences in MUS between men and women in different age groups were analyzed using two-factor analysis of variance. It revealed no interaction between sex and different age groups on MUS. The main effect analysis showed that the effects of different age groups on MUS were statistically significant. Based on pairwise comparative analysis, the MUS score in the low age group was lower than that in the middle age group. To clarify a nonlinear relationship between age and MUS, threshold effect analysis was conducted. The results indicated that the piecewise linear regression model could better depict the relationship between age and MUS. The inflection point was at the age of 60 years. Before the age of 60 years, MUS increased with age. No significant correlation existed between age and MUS after the age of 60 years. To understand the influencing factors of MUS, the intermediary effect model was analyzed using MUS as the dependent variable, psychological stress as the independent variable, resilience as mediator variable M1, perceived social support as mediator variable M2, and depression as mediator variable M3. Resilience, perceived social support, and depression had significant mediator effects on the effects of psychological stress on MUS with a total indirect effect of 69.81%.

**Conclusion:**

The middle age group had greater MUS than the low age group. Before the age of 60 years, MUS increased with increasing age. Women had more severe MUS than men. Resilience, perceived social support, and depression had significant mediating effects on the effects of perceived stress on MUS. These findings suggest that clinicians should make more comprehensive and detailed evaluations and timely intervention for middle-aged and female patients. Improving psychological resilience and social support can reduce the impact of psychological stress on MUS. Therefore, psychotherapy and multidisciplinary comprehensive treatment are very important for patients is very important for patients.

## Introduction

Medically unexplained symptoms (MUS) is one of the most common problems encountered by clinical departments, which was first proposed by Slavney and Teitelbaum (1). The meaning of MUS is the somatic symptoms that cannot be reasonably explained by the pathological structural changes and pathophysiological abnormalities of biomedicine (2). Clinical workers are also used to calling MUS as nonspecific symptom, functional somatic symptom or neurosis.Based on findings of a survey conducted in certain developed countries (3), approximately one-third of patients in the outpatient department of general hospitals have MUS. The Diagnostic and Statistical Manual of Mental Disorders, Fourth Edition (DSM-IV) and the 10th edition of the International Classification of Diseases (ICD-10) classify MUS as a “somatoform disorder.” In actual clinical diagnosis and treatment, doctors and patients are often dissatisfied (4). The DSM-5 replaced somatoform disorder with “somatic symptoms and related disorders.” This classification no longer emphasizes that somatic symptoms cannot be explained medically, but highlights poor adaptation to somatic symptoms (5). ICD-11 issued by the World Health Organization also replaced somatoform disorder with “Disorders of bodily distress or bodily experience” (6) in order to integrate the previous diagnostic system. Therefore, for patients with somatic symptoms in outpatient departments of general hospitals, compared with the previous dualism-based view, their symptoms are not considered a medical problem or a psychological problem (7), but the psychosomatic diseases in which the two interact are considered. The patient health questionnaire physical symptoms scale (PHQ-15) is a widely used physical symptoms evaluation scale with good reliability and validity (8, 9), and has been translated into many languages.

Psychosomatic diseases can be divided into three stages: psychosomatic reactions, psychosomatic disorders (with functional lesions), and psychosomatic diseases (with somatic organic lesions) (10). Epidemiological data on psychosomatic diseases in China and globally show that the incidence rate of psychosomatic diseases is approximately 22–35%. Early intervention in psychosomatic diseases is very important, especially during the stage of a psychosomatic reaction and psychosomatic disorder so as to avoid further aggravation of psychosomatic diseases. Patients for whom a physical examination cannot partially or fully explain their symptoms often have depression, anxiety, and other problems, which complicates the disease. A study (11) showed that 50% of patients with depression have a variety of MUS, and 35% of patients with MUS have depression. The physical symptoms of psychosomatic patients and psychological stress are linked. For people who are susceptible to stress, even mild to moderate stress may lead to psychosomatic diseases (12). Psychological resilience and social support provide a type of protection for psychosomatic patients. Psychological resilience is defined by the American Psychological Association as a good adaptation process for individuals to face adversity, trauma, tragedy, threat, or other major pressures (i.e., the ability to rebound from difficult experiences). Psychological resilience has multidimensional characteristics, which changes with background, time, age, sex, culture, and different living environments (13). Social support is the object or resource that an individual can turn to when facing pressure, including help from family, friends, and society. Compared to objective social support, the social support that individuals subjectively experience is more related to emotional support and inner satisfaction (i.e., perceived social support). Some studies have shown that perceived social support is more important to patients than objective social support (14). The more a person experiences being respected, supported, and understood emotionally, the higher the person's inner satisfaction is, and the stronger their ability to resist pressure.

A cross-cultural study (15) conducted by the World Health Organization on the primary health care sector in 14 countries revealed that individuals aged ≥45 years tended to have a higher risk of somatisation than patients aged 31–44 years. Sex-specific somatic symptoms have been reported (16, 17); in general, women usually report more intense and frequent somatic symptoms. The present study aimed to understand whether age and sex are risk factors for MUS. Patients with MUS should be comprehensively evaluated as soon as possible after presentation to understand the possible influencing factors and to ensure timely intervention, especially in the psychosomatic reaction and psychosomatic disorder stages, so as to avoid further aggravation of psychosomatic diseases.

## Method

### Participants

This is a cross-sectional study. Convenient sampling was used to investigate outpatients in the Medical Psychology Department of Chinese PLA (People's Liberation Army) General Hospital (< city>Beijing < /city>, China) from April 1, 2021 to August 9, 2021. The participants were non-military civilians. The inclusion criteria were (1) age ≥18 years; (2) visiting the Department of Medical Psychology for the first time; and (3) the presence of somatic symptoms (Patient Health Questionnaire-15 [PHQ-15]≥1). The exclusion criteria were (1) the presence of psychotic symptoms, (2) patients with no insight and self-evaluation ability; and (3) somatic symptoms caused by physical diseases. All participants provided written informed consent. The protocol was approved by Chinese PLA General Hospital Ethics Committee.

### Design

An exploratory observational study design was employed. The doctors in the psychological clinic are qualified as psychiatrists and are responsible for consultation and mental examination. Patients were recruited into the study after meeting the inclusion criteria.The enrolled patients provided general information and completed the self-assessment scale in the integrated psychosomatic comprehensive assessment system (IPS) on their mobile phone. Technical problems and questionnaire questions in this process were addressed by psychometrists who had been systematically trained; all information provided was kept confidential.Psychometrists have professional background in psychology and are skilled in various psychological measurement tools.

### Measures

#### Patient health questionnaire-15

Körber et al. (18) demonstrated that the PHQ-15 has good reliability and validity and is suitable for screening for and evaluating the severity of MUS. It contains 15 items, which primarily measure the patients' MUS in the preceding 4 weeks before presentation. Symptom severity is divided into three grades: 0 (no distress), 1 (some distress), and 2 (a lot of distress). The total score range is 0–30 points, in which 0–4 indicates no MUS; 5–9 mild MUS; 10–14 moderate MUS; and ≥15 points severe MUS. When used to predict the diagnosis of somatisation disorder, the 10-point threshold of the PHQ-15 was determined to be the best value with a sensitivity of 80.2% and a specificity of 58.5%.

#### Patient health questionnaire-9

The PHQ-9 scale (19) is used to screen and evaluate depressive symptoms. It contains nine items, each with a score of 0 (not at all) to 3 (nearly every day). The total score range is 0–27 points, where 0–4 points indicates no depression; 5–9 points mild depression; 10–14 points moderate depression; 15–19 points moderate to severe depression; and 20–27 points severe depression.

#### Insomnia severity index

The Insomnia Severity Index (ISI) (20) is a reliable and effective tool for screening insomnia and evaluating its degree; it includes seven items. It contains five grades from 0 to 4. The higher the score, the more serious the symptoms of insomnia; 0–7 points indicates insomnia without clinical significance; 8–14 points subclinical insomnia; 15–21 points moderate clinical insomnia; and 22–28 points severe clinical insomnia.

#### Perceived stress scale-10

The Perceived Stress Scale-10 (PSS-10) is the most widely used stress perception assessment tool. The scale is a self-assessment tool compiled by Cohen et al. in 1983 (21). The 10-item version of the PSS-10 has been proven to have good consistency in large-scale community research in China (22). The PSS-10 is used to evaluate the uncontrollable, unpredictable, or overloaded situations in an individual's own life. Each item is graded from 0 (never) to 4 (very common). The scale is divided into 0–40 points for the total score of each item. The higher the score, the higher the perceived stress level.

#### Perceived social support

The Perceived Social Support (PSSS) (23) contains 12 items that can be carefully evaluated from three dimensions: family support, friend support, and other support. Each item adopts the 1–7 level scoring method. The higher the total score, the higher an individual's social support. A total score of 12–36 is considered low support, 37–60 medium, and 61–84 high.

#### Connor–davidson resilience scale

The Connor–Davidson Resilience Scale (CD-RISC) scale (24) contains 25 items, each of which is evaluated on a 5-point scale. The scores 0 to 4 indicate “not at all,” “rarely,” “sometimes,” “often,” and “almost always,” respectively. The total score of the scale is 0–100. The higher the score, the higher is the psychological resilience.

### Statistics

The data were statistically analyzed using SPSS 22.0 (IBM Corp., Armonk, NY, USA). The continuous variables are presented as the mean ± the standard deviation. Classification variables are expressed as the count and percentage. The differences between different age groups and different sexes were evaluated using the chi-squared test, one-way analysis of variance (ANOVA), and the independent samples *t*-test. Two-factor ANOVA was used to analyse differences in MUS between men and women in different age groups. The nonlinear relationship between age and MUS was visualized using a smoothed curve fitting (25). After visualizing the nonlinear relationship between age and MUS with a smoothed curve, for those with a potential nonlinear relationship, the piecewise regression model was used for fitting and the log likelihood ratio test was used to determine whether the nonlinear relationship was significant and whether a significant inflection point existed. The inflection point of the connecting line segment was determined based on the maximum likelihood value given by the piecewise regression model. In the piece-wise linear regression model, the turning point was determined using the trial-and-error method: firstly, selecting turning points according to a pre-defined interval and then choosing the turning point that gave the maximum model likelihood. In addition, a log-likelihood ratio test was conducted to compare the one-line linear regression model with the two-piecewise linear model. A 2-tailed *P* < 0.05 was considered to be statistically significant in analyses.The influencing factors of MUS were analyzed using the chain-mediated effect model in SPSS 22.0 (Process 3.4). Bootstrapping was used to test the significance of mediating effects.

## Results

Seven hundred forty-six patients completed the integrated psychosomatic assessment of the IPS and included 467 (62.6%) men and 279 (37.4%) women. The youngest patient was 18 years old and the oldest patient was 79 years old. The average age was 33.21 ± 11.55 years. The patients were divided into three age groups: 18–25 years old (low age group), 26–44 years old (middle age group), and >45 years old (high age group). The PHQ-15 score used to measure MUS was <10 (i.e. mild physical symptoms) in 230 (30.8%) patients and ≥10 (i.e. moderate and severe physical symptoms) in 516 (69.2%) patients.

Differences in sex and scale scores among the different age groups are shown in Table 1. The sex ratios of the three groups were significantly different. There were significant differences in PHQ-9, PSS-10, PSSS and CD-RISC among different age groups.No significant difference existed in PHQ-9, PSS-10, PSSS and CD-RISC scores in the middle and low age groups, PHQ-9 and PSS-10 scores were higher than those in the high age group, CD-RISC and PSSS scores were lower than those of the high age group in further pairwise comparison. PHQ-15 scores were higher in the middle age group than in the low age group. No significant difference existed between the two groups and the high age group.

**Table 1:** Comparison of different age groups.

	**18–25 (*n* = 224)**	**26–44 (*n* = 390)**	**45 (*n* = 132)**	**F/χ^2^**	***p*-value**
Sex				33.819	^**^
Male	164 (73.21%)	247 (63.33%)	56 (42.42%)		
Female	60 (26.79%)	143 (36.67%)	76 (57.58%)		
PHQ-15	11.95 ± 6.284	13.63 ± 6.778	13.42 ± 6.614	4.818	^**^
PHQ-15 Unweighted marginal mean*	12.071 ± 0.498 95%CI (11.092–13.049)	13.695 ± 0.347 95%CI (13.013–14.376)	13.307 ± 0.582 95%CI (12.165–14.449)	3.601	0.028
PHQ-15				1.21	0.546
<10	75 (33.48%)	114 (29.23%)	41 (31.06%)		
≥10	149 (66.52%)	276 (70.77%)	91 (68.94%)		
PHQ-9	14.51 ± 6.758	13.90 ± 6.635	11.89 ± 6.781	6.612	^**^
ISI	13.86 ± 6.816	14.80 ± 7.060	14.40 ± 7.057	1.282	0.278
PSS-10	23.46 ± 3.899	23.91 ± 3.958	22.01 ± 4.973	16.537	^**^
PSSS	45.42 ± 17.680	47.27 ± 16.062	53.03 ± 15.702	9.15	^**^
CD-RISC	38.19 ± 16.525	39.88 ± 16.514	47.39 ± 15.393	14.222	^**^

PHQ-15, Patient Health Questionnaire-15; PHQ-9, Patient Health Questionnaire-9; ISI, Insomnia Severity Index; PSS-10, Perceived Stress Scale-10; PSSS, Perceived Social Support; CD-RISC, Connor–Davidson Resilience Scale.

** p < 0.01.

Sex differences in age and scales are shown in Table 2. The average age and PSSS score of female patients were higher than those of male patients, but more female patients had moderate and severe physical symptoms.

**Table 2:** Comparison of different sexes.

	**Male patients (*n* = 467)**	**Female patients (*n* = 279)**	**T/χ^2^**	***p*-value**
Age	30.97 ± 9.88	36.97 ± 13.08	−6.616	^**^
PHQ-15	12.76 ± 6.87	13.63 ± 6.20	−1.785	0.075
PHQ-15			6.889	^**^
<10	160 (34.26%)	70 (25.09%)		
≥10	307 (65.74%)	209 (74.91%)		
PHQ-9	13.59 ± 6.85	13.96 ± 6.58	−0.721	0.471
ISI	14.79 ± 7.18	13.86 ± 6.65	1.772	0.077
PSS-10	23.56 ± 4.05	23.24 ± 4.42	1.021	0.308
PSSS	39.43 ± 16.82	42.83 ± 16.07	−2.715	^**^
CD-RISC	48.59 ± 17.37	46.30 ± 15.38	1.878	0.061

PHQ-15, Patient Health Questionnaire-15; PHQ-9, Patient Health Questionnaire-9; ISI, Insomnia Severity Index; PSS-10, Perceived Stress Scale-10; PSSS, Perceived Social Support; CD-RISC, Connor–Davidson Resilience Scale.

** p < 0.01.

The results revealed no interaction between sex and the different age groups (*F*[2, 740] = 0.321, *p* = 0.726, partial η^2^ = 0.001). The main effect analysis revealed that the effects of different age groups on PHQ-15 scores were statistically significant (*F*[2, 740] = 3.601, *p* = 0.028, partial η^2^ = 0.01). The main effect results of different age groups were analyzed using a pairwise comparison. The PHQ-15 score of the low age group was lower than that of the middle age group (95% CI, 0.35–3.01; *p* < 0.01) (Table 1).

**Figure 1:**
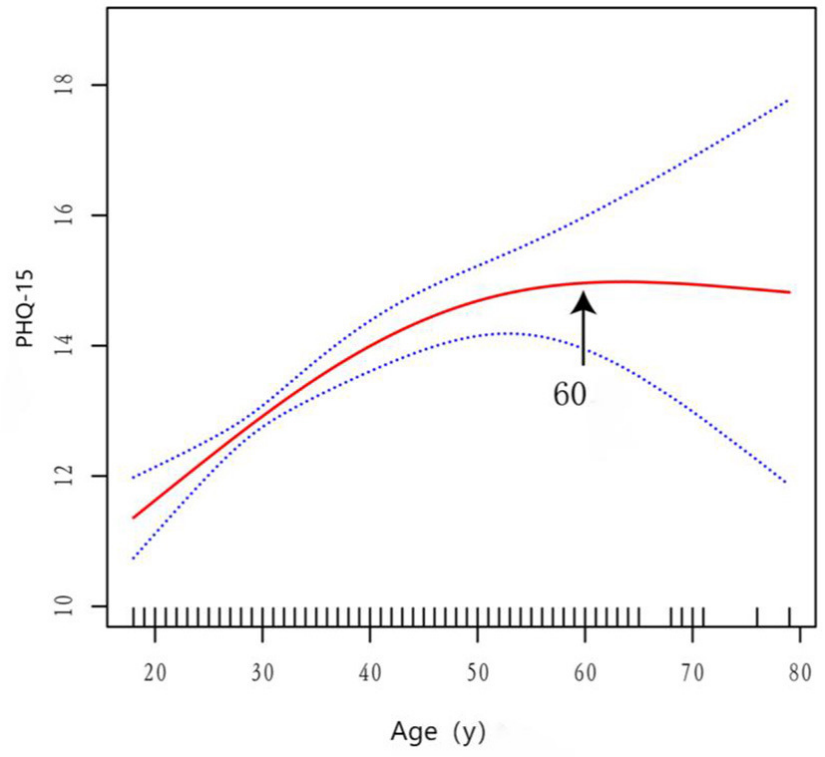
Multivariable adjusted smoothed spline curve of age and MUS.

To explore whether a nonlinear relationship existed between age and MUS, smoothed curve fitting analysis was conducted for visualization (Figure 1). The slope of the smoothed curve between age and MUS changed, which suggested the presence of an inflection point. To determine whether a significant inflection point in the smoothed curve existed between age and MUS, threshold effect analysis was conducted, as shown in Table 3. The results demonstrated that the piecewise linear regression model could better describe the relationship between age and MUS. The inflection point was at 60 years of age: before 60 years of age, MUS increased with increasing age; after 60 years old, the correlation between age and MUS was not statistically significant. The arrows in Figure 1 indicate significant inflection points.

**Table 3:** Threshold effect analysis of age and MUS.

**Outcome variable**	**MUS**	
	**β (95%CI)**	***p*-value**
Model 1: Linear regression	0.09 (0.06, 0.12)	<0.001
Model 2: Piecewise linear regression		
Inflection point K	60
< K	0.10 (0.07, 0.14)	<0.001
≥K	−0.21 (−0.50, 0.08)	0.158
Effect difference at both ends	−0.31 (−0.61, −0.01)	0.042
Predicted value of the equation at the inflection point	14.64 (13.32, 15.96)	
Log likelihood ratio tests	0.041	

**Figure 2:**
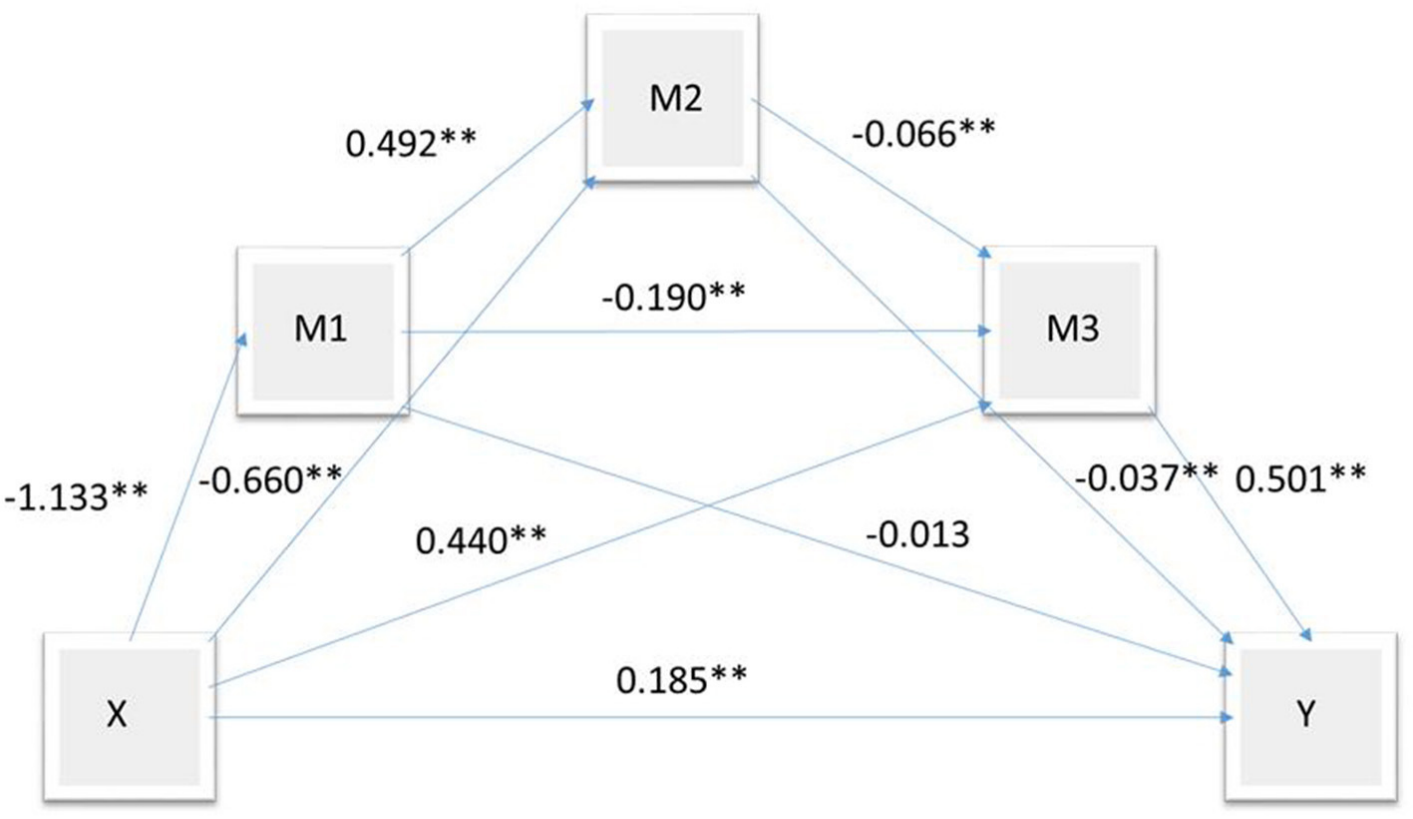
Path map of psychological stress on MUS; Y indicates MUS; X, psychological stress; M1, resilience; M2, perceived social support; M3, depression. **p < 0.01.

To understand the factors influencing MUS, we conducted a mediation effect model analysis. MUS was the dependent variable, and psychological stress was the independent variable. Psychological resilience was mediation variable M1, perceived social support was mediation variable M2, and depression was mediation variable M3. Regression analysis revealed that psychological resilience, perceived social support, and depression had significant mediating effects on the effects of psychological stress on MUS (Table 4). The total indirect effect was 69.81% (Table 5). Figure 2 presents the path diagram of the impact of perceived stress on somatic symptoms.

**Table 4:** Regression analysis between variables.

	**Regression equation**		**Overall fitting index**		**Significance of the regression coefficient**	
**Result variable**	**Predictive variable**	***R***	***R*** ^**2**^	***F***	***β***	***t***
Psychological resilience	Psychological stress	0.285	0.081	65.543	−1.133	−8.096**
Perceived social support	Psychological stress	0.565	0.319	174.026	−0.660	−5.272**
	Psychological resilience				0.492	15.656**
Depression	Psychological stress	0.712	0.507	254.259	0.440	9.977**
	Psychological resilience				−0.190	−15.175**
	Perceived social support				−0.066	−5.236**
MUS	Psychological stress	0.646	0.417	132.441	0.185	3.684**
	Psychological resilience				−0.013	−0.833
	Perceived social support				−0.037	−2.676**
	Depression				0.501	12.738**

** p < 0.01.

**Table 5:** Mediation effects of psychological resilience, perceived social support, and depression on the relationship between psychological stress and MUS.

	**Effect value**	**Boot standard error**	**BootCI low limit**	**BootCI upper limit**	**Relative mediating effect**
Total indirect effect	0.428	0.040	0.352	0.5109	69.81%
Indirect effect 1	0.015	0.018	−0.023	0.0504	2.36%
Indirect effect 2	0.024	0.010	0.007	0.0467	3.98%
Indirect effect 3	0.220	0.032	0.162	0.2867	35.91%
Indirect effect 4	0.021	0.009	0.005	0.0398	3.36%
Indirect effect 4	0.108	0.018	0.074	0.1455	17.59%
Indirect effect 6	0.022	0.006	0.011	0.0359	3.59%
Indirect effect 7	0.019	0.005	0.010	0.0293	3.02%

*Indirect effect 1: X → M1 → Y; Indirect effect 2: X → M2 → Y; Indirect effect 3: X → M3 → Y; Indirect effect 4: X → M1 → M2 → Y; Indirect effect 5: X → M1 → M3 → Y; Indirect effect 6: X → M2 → M3 → Y; and Indirect effect 7: X → M1 → M2 → M3 → Y*.

## Discussion

This cross-sectional study had several findings: (1) most patients had moderate and severe MUS and a high proportion of patients had severe MUS; (2) MUS was higher in the middle age group than in the low age group; (3) before the age of 60 years, MUS increased with increasing age; (4) more male patients than female patients were young to middle aged, whereas more female patients were in the high age group; (5) women had more severe MUS than men; and (6) psychological resilience, perceived social support, and depression had significant mediating effects on the effects of psychological stress on MUS.

Our study demonstrated that MUS in the middle age group was higher than in the low age group, and no significant difference existed between these two groups and the high age group. These findings differ from the findings of a World Health Organization study (15) conducted in 14 countries, which indicated that the risk of somatisation is higher in people >45 years of age. A possible reason for this difference is that we chose patients from the psychological clinic of a tertiary level A hospital in Beijing, China. In China's first-tier cities, 18- to 25-year-old individuals are facing the pressure of entering school and employment, and 26- to 44-year-old individuals have increased family, career, and social and psychological pressures, whereas, in most first-tier cities, individuals >45 years old have successful careers and happy families. Our research also confirmed this. Depression and psychological stress in the low and middle age groups were higher than those in the high age group, whereas psychological resilience and perceived social support were lower than those of the high age group. The threshold effect analysis of the relationship between age and MUS revealed that MUS increased with age until 60 years, but no significant correlation existed between MUS and age after 60 years.

The analysis of different sexes revealed significant differences in the severity of MUS between men and women. More women than men experienced moderate and severe MUS. Consistent with the present study, sex-specific somatic symptoms have been reported. In general, women usually report more intense and frequent somatic symptoms. The severity of MUS also predicted the level of mental health (26). As in the present study, the middle and low age groups contained more men than women, whereas the high age group contained more women. A study (27) conducted in the outpatient department of Guangzhou General Hospital in China also showed that female sex, depression, and anxiety were the main risk factors of serious physical symptoms. Research has also shown opposing findings in terms of sex; the findings of one study (28) in adolescents showed that male individuals had more psychosomatic symptoms. Another study (29) on the correlation between sex, age, and physical symptoms also demonstrated that men had greater severity of physical symptoms. Findings in one study (30) revealed sex differences in somatic symptoms; fewer women than men went to a hospital for physical examination and diagnosis. The reasons for the inconsistencies in these studies may be that the applied somatic symptom measurement scale differed, the items of somatic symptoms are different between men and women, women are more sensitive to pain, and women are more likely to report gastrointestinal discomfort than men.

Some studies (18, 31) demonstrated that a correlation exists between the number and severity of MUS, negative psychological symptoms, and functional characteristics. The present study supports this finding in the further analysis of the influencing factors of somatic symptoms. Many studies indicated that psychological stress can lead to depression and MUS. A study (32) of 604 patients in a psychosomatic clinic showed that the number of MUS is associated with psychological stress and the severity of depression. Investigators from different medical disciplines believe that somatic symptoms are associated with changes in hypothalamic pituitary adrenal function, an imbalance in vagus sympathetic tone and the upregulation of immune and inflammatory function, and an enhanced response to threatening stimuli so as to promote the subjective experience of somatic symptoms (33). Somatic symptoms reflect disorders of the stress system. Psychological stress affects the pathogenesis of physical diseases through negative emotions such as depression or anxiety. Exposure to chronic stress is considered the most harmful because it is most likely to lead to long-term or permanent changes in emotional, physiological, and behavioral responses, thus affecting susceptibility and the course of physical diseases (34, 35). The analysis of the mediating effect model of physical symptoms caused by psychological stress revealed that psychological resilience, perceived social support, and depression all have mediating effects on MUS caused by psychological stress and that psychological resilience and perceived social support have a protective role in the occurrence of physical symptoms. A study (36) on resilience and mental health showed that psychological resilience depends on an individual's thoughts, emotions, and behaviors and depends on available and obtained cultural resources, such as the social environment. The intervention in multiple systems and fields of psychological resilience is more likely to establish psychological resilience to properly deal with serious or long-term adversity. One study (37) on the role of perceived social support in depression and somatic symptoms among college students showed that the ability to obtain emotional support could predict depression and physical symptoms.

Studies have confirmed that the outpatient and inpatient costs of patients with MUS are high (38) and that direct and indirect expenditures are increased, especially for patients with severe MUS (31). The present study and a previous study (39) demonstrated that MUS were closely associated with psychosocial factors. For the treatment of MUS, research emphasizes the effectiveness of psychosocial therapy (especially cognitive behavioral therapy) and the importance of multidisciplinary management (40). Therefore, comprehensive psychological evaluation can provide support for a patient's psychotherapy.

There are a few limitations. First, at present, there are still great differences on the clinical definition, diagnostic classification, pathological mechanism and clinical treatment of MUS. Our study included participant with MUS as long as there was one or more somatic discomfort symptoms that could not be explained by physical diseases. Secondly, we did not evaluate the duration of MUS, its impact on patients' daily life and the distress it brought to patients, so we did not make a clear diagnosis for the participants according to the ICD or DSM diagnostic system. We made a cross-sectional evaluation and did not continue to follow up.

The findings of the present study suggest that clinicians should make a more comprehensive and detailed evaluation and timely intervention for middle-aged and female patients. Our study has identified for the first time that MUS increased with age until 60 years, but no significant correlation existed between MUS and age after 60 years. Improving psychological resilience and social support can reduce the impact of psychological stress on physical symptoms. Therefore, psychotherapy is very important for patients. CBT (Cognitive Behavioral Therapy) and MBCT (Mindfulness Based Cognitive Therapy) have been proved feasible and effective intervention methods by many studies (41, 42). In terms of drug treatment, as patients with MUS are often accompanied by symptoms such as anxiety and depression, antidepressant drugs are usually given to patients to help them relieve pain and restore social function. Before visiting the psychological clinic, patients with MUS often repeatedly visit other outpatient clinics in general hospitals, so multidisciplinary consultation and liaison is beneficial.

Subsequent studies can follow up these patients, including the definite diagnosis, treatment and prognosis. The clinical effects of different treatment methods and the clinical outcomes of patients of different sex and age were compared.

## Data availability statement

The datasets presented in this article are available on request to the authors. Requests toaccess the datasets should be directed to xiaojie8706@163.com.

## Ethics statement

The studies involving human participants were reviewed and the protocol was approved by Chinese PLA General Hospital Ethics Committee. The patients/participants provided their written informed consent to participate in this study.

## Author contributions

All authors listed have made a substantial, direct, and intellectual contribution to the work and approved it for publication.

## Funding

This study was supported by Capital's Funds for Health Improvement and Research (CFH2018-2-5011) and Young Talents Project of Military Medical Fund (QNC19003).

## Conflict of interest

The authors declare that the research was conducted in the absence of any commercial or financial relationships that could be construed as a potential conflict of interest.

## Publisher's note

All claims expressed in this article are solely those of the authors and do not necessarily represent those of their affiliated organizations, or those of the publisher, the editors and the reviewers. Any product that may be evaluated in this article, or claim that may be made by its manufacturer, is not guaranteed or endorsed by the publisher.

## Abbreviations

ANOVA: analysis of variance
MUS: medically unexplained symptoms
CD-RISC: Connor–Davidson Resilience Scale
ISI: Insomnia Severity Index
PHQ-9: Patient Health Questionnaire-9
PHQ-15: Patient Health Questionnaire-15
PSS-10: Perceived Stress Scale-10
PSSS: Perceived Social Support.
